# Closed trail distance in a biconnected graph

**DOI:** 10.1371/journal.pone.0202181

**Published:** 2018-08-31

**Authors:** Vaclav Snasel, Pavla Drazdilova, Jan Platos

**Affiliations:** Department of Computer Science, Faculty of Electrical Engineering and Computer Science, VŠB - Technical University of Ostrava, 17. listopadu 15/2172, 708 33 Ostrava, Czech Republic; Bar-Ilan University, ISRAEL

## Abstract

Graphs describe and represent many complex structures in the field of social networks, biological, chemical, industrial and transport systems, and others. These graphs are not only connected but often also k-connected (or at least part of them). Different metrics are used to determine the distance between two nodes in the graph. In this article, we propose a novel metric that takes into account the higher degree of connectivity on the part of the graph (for example, biconnected fullerene graphs and fulleroids). Designed metric reflects the cyclical interdependencies among the nodes of the graph. Moreover, a new component model is derived, and the examples of various types of graphs are presented.

## Introduction

More interconnected parts of graphs play an essential role in the social and natural sciences. The formalization of the term “more connected part” can be defined in many ways. In this article, we focus on generalizing biconnected components of a graph and we define a novel metric that considers higher degree of connectivity on the part of the graph. Biconnected components of the graph do not allow good scalability, and their definition is complicated for weighted graphs. Our approach is based on the cycle length limit in the definition of biconnected components. The first work devoted to the study of cycles of limited length is [1, 2].

Topological data analysis often uses tools developed in algebraic topology [3–5]. Cycles play a significant role in algebraic topology. For example, Poincaré’s theorem of duality [6] shows that the homology group *H*_*n*_(*M*, *Z*_2_) is a space with the inner product above the field *Z*_2_ where the inner product is defined as an intersection index. In the case that *M* is a continuous manifold, then any homology class *x* ∈ *H*_1_(*M*, *Z*_2_) can be represented by a closed curve *γ* ⊂ *M*. In this case, the intersection index *x* ⋅ *x* becomes zero when and only when a small surrounding of the curve *γ* is orientable.

Cycles with limited length play an essential role in the application of algebraic topology [3, 4]. When calculating the topological properties of data, it is necessary to look for cycles of limited length [7, 8]. Cyclical structures are also very often found in materials research. For example, fullerenes form long cycles [9], in which topological properties play an important role. Complex and social networks are another field in which cyclic structures appear [10].

The partitioning of large complex networks is a challenging task. Such networks are used as a representation of proteins, chemical compounds, co-author networks, social networks, etc. The classical partitioning methods have problems with densely connected subgraphs that cannot be partitioned easily. A list of the largest biconnected component in the selected network was published by Leskovec [11].

In the case of protein interaction networks in computational biology, the authors in [12] found vertices that are articulation points (determined by the computing of biconnected components), but they have a low degree and, therefore, they are unlikely to be essential to the network. In [13] the authors found the biconnected components that enabled further analysis.

**Fig 1:**
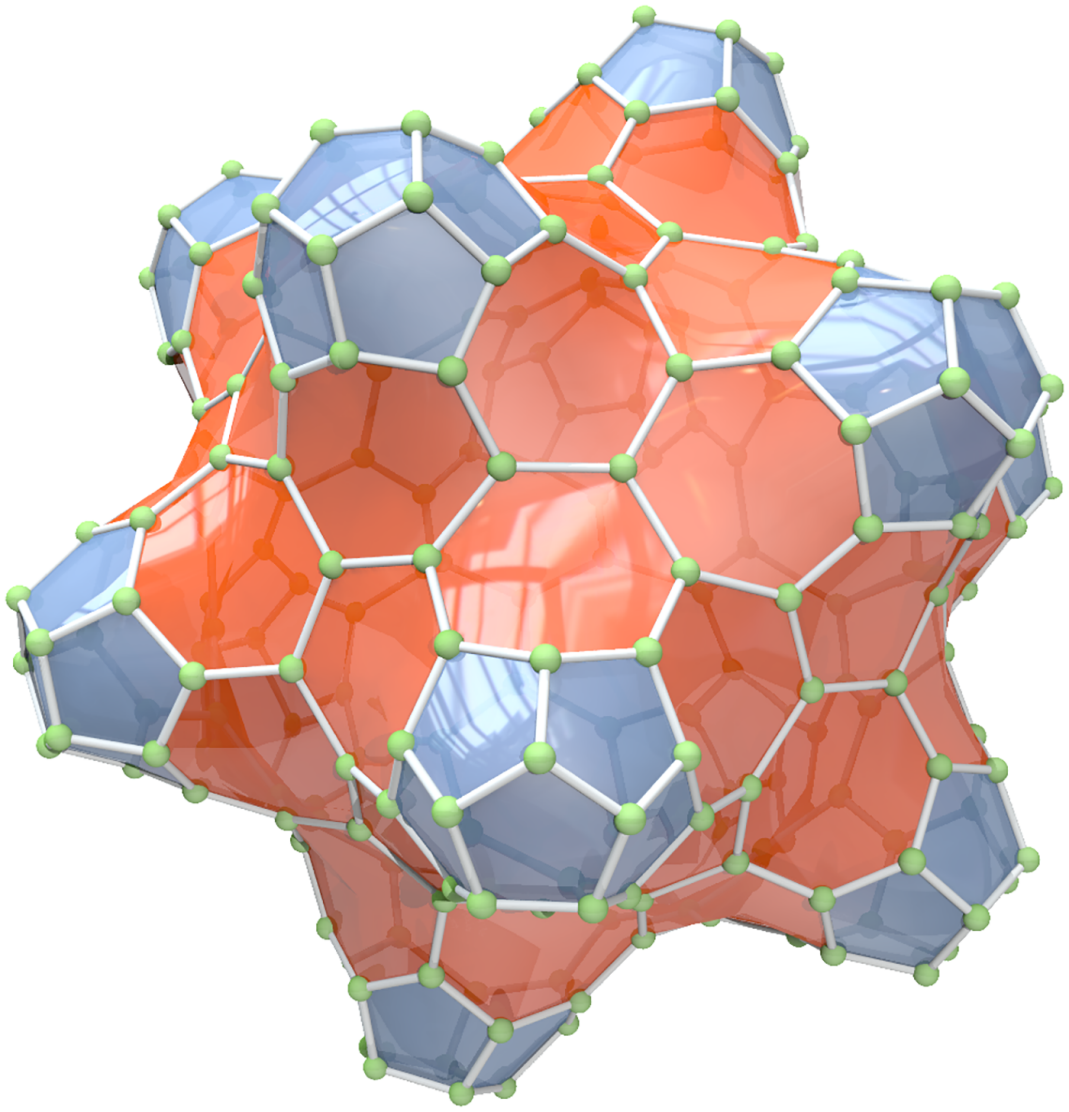
Graph of a fulleroid *C*_260_ − *I*[5, 7] (The image was provided by Peter Schwerdtfeger, Lukas N. Wirz and James Avery under a CC BY license).

Molecular topology [14] is another area where topological and metric distances are used in graphs representing molecules. The fullerenes are cage-like, hollow molecules of pseudo-spherical symmetry consisting of pentagons and hexagons only, resulting in a trivalent polyhedron with precisely three edges (bonds) joining every vertex occupied by carbon [9]. In graph theoretical terms, fullerenes belong to the class of cubic, planar, three-connected, and simple graphs, see Fig 1. The authors of [15] give an overview of some graph invariants that can possibly correlate with the stability of a fullerene molecule.

Fullerenes have been the subject of intense research for their unique physical, chemical, and biological properties and for their technological applications, especially in materials science, electronics, nanotechnology, and medicine [16–19].

The measuring of the distances between two nodes in a graph is a difficult task. The standard measure for this distance is the shortest path between two nodes in a graph [20, 21]. Another way is the expected lengths of random walks on the graph, which can be used to derive the commute time distance [22]. The authors of [23] examine generalized distances on graphs that interpolate, depending on a defined parameter, between the shortest path distance and the commute time or resistance distance. Variants of node distances are described in detail in [24–27].

In addition to the node distance measure, the quality of the components (partitions) is also measured by means of several approaches. In 2014, a metric that measured the quality of communities according to the number of 3-cycles split across the communities was published [28]. The idea is based on the four types of directed triangles that contain cycles. These triangles are used to identify communities in directed networks. In [29], a measure that integrates both the concept of closed walks and clustering coefficients to replace the edge betweenness in the divisive hierarchical clustering algorithm (the Girvan and Newman method) was published. Levoranto et al. [30] used the strongly *p*-connected components for community detection in oriented networks. Community detection in undirected graphs is different. One of the major and well-known approaches uses the union of cliques to define a community [31]. Edaschery et al. [32] defined *distance-k clique* of a graph *G* = (*V*, *E*) as a subgraph of *G* with a diameter *k*. The authors use these *distance-k cliques* for a clustering.

Our approach defines a new type of metric in a graph based on “cyclical distances“. This metric is based on the definition of a biconnected component. The distance between two vertices in the graph is defined as the length of the shortest closed trail that contains these two vertices. The distance defined in this way allows straightforward generalization for weighted graphs and also allows scalability.

In this article, we define a new measure on an undirected connected graph without bridges for the measurement of distances using cyclic subgraphs. This measure satisfies the metric properties. Our innovative measure may be used to define a new type of components that highlight the locally connected subgraphs. Moreover, these components are not based on the biconnectivity property and, therefore, are able to partition densely connected biconnected components easily.

We will first introduce the terminology and the notation which we use in the article. In the next section, we define the new distance in biconnected undirected graphs, and we describe some properties of this distance. In conclusion, we discuss the advantages and limitations of the defined distance.

## Terminology and notation

In this section, knowledge of graph theory will be required. The definitions of the following terms were taken from [33]:

A *loop* is an edge (directed or undirected) that joins a single endpoint to itself. A *walk* on a graph is an alternating series of vertices and edges

$$\begin{array}{c} W\left(v_{\left(0\right)},v_{\left(k\right)}\right)=v_{\left(0\right)}e_{\left(1\right)}v_{\left(1\right)}e_{\left(2\right)}\ldots v_{\left(k-1\right)}e_{\left(k\right)}v_{\left(k\right)} \end{array}$$

such that for *j* = 1, …, *k* the vertices *v*_(*j*−1)_ and *v*_(*j*)_ are the end points of the edge *e*_(*j*)_. A *closed walk* is a walk where the initial vertex is also the final vertex. The *length of a walk* is the number of edges in this walk. We will denote the length of a walk *W*(*u*, *v*) as |*W*(*u*, *v*)|. A *trail* is a walk in which no edge occurs more than once. A *closed trail* (*circuit*) is a closed walk with no repeating edges. We will denote a closed trail which contains the vertices *u*, *v* as

$$\begin{array}{c} CT\left(u,v\right)=ue_{\left(1\right)}v_{\left(1\right)}e_{\left(2\right)}\ldots v\ldots e_{\left(k\right)}u. \end{array}$$

A *path* is a walk in which no edge or internal vertex occurs more than once (a trail in which all the internal vertices are distinct). We will denote a path with an initial vertex *u* and a final vertex *v* as *P*(*u*, *v*). A *cycle* is a closed path with a length at least one. We will denote the closed path containing *u*, *v* as *CP*(*u*, *v*). A *clique* is a subgraph where each node is adjacent to every other node. A *planar graph* is a graph that can be drawn on a sphere or a plane with no edge crossings.

A *connected* graph is a graph such that between every pair of vertices there exists a walk. A graph is called *k-connected* if the removal of fewer than *k* vertices leaves neither a non-connected graph nor a trivial one. A *component* of a graph is a maximal connected subgraph. An edge *e* is a *bridge* of the connected graph *G* if {*e*} is a disconnecting edge-set of *G*. An *articulation* is a vertex of a graph whose removal increases the number of components. A *biconnected* graph is a connected and “nonseparable” graph, meaning that if any vertex were to be removed, the graph would remain connected. Therefore a biconnected graph has no articulation vertices.

The property of being *2-connected* is equivalent to biconnectivity, with the caveat that the complete graph of two vertices is sometimes regarded as biconnected but not 2-connected. This property is especially useful in maintaining a graph with a two-fold redundancy, to prevent disconnection upon the removal of a single edge (or connection).

A *biconnected component* (or 2-connected component) is a maximal biconnected subgraph.

### Equivalent characterizations of biconnectivity

Let *G* = (*V*, *E*) be a simple undirected graph (loop-less, no multiple edges) that contains at least three points. Each of the following statements is equivalent to that G is biconnected

1. for every *v*_1_, *v*_2_ ∈ *V* there is a circuit of G containing *v*_1_ and *v*_2_,
2. *E* ≠ ∅ and for every *v* ∈ *V* and *e* ∈ *E* there is a cycle of G containing *v* and *e*,
3. *G* has no isolated vertices and for every *e*_1_, *e*_2_ ∈ *E* there is a cycle containing *e*_1_ and *e*_2_,
4. for every *v*_1_, *v*_2_, *v*_3_ ∈ *V* there is a path from *v*_1_ to *v*_2_ containing *v*_3_,
5. for every *v*_1_, *v*_2_, *v*_3_ ∈ *V* there is a path from *v*_1_ to *v*_2_ not containing *v*_3_,
6. *E* ≠ ∅ and for every *v*_1_, *v*_2_ ∈ *V* and *e* ∈ *E* there is a path from *v*_1_ to *v*_2_ containing *e*.

The shortest path metric (*d*_*sp*_) [25] is the one most commonly used to determine the distance between vertices of the graph. It is a metric on the vertex-set *V* of a connected graph *G* = (*V*, *E*), defined, ∀*u*, *v* ∈ *V*, as the length of the shortest path (*P*(*u*, *v*)) in *G*. This metric does not affect greater coherence between vertices in the biconnected graph. Our goals in this article are to define a new metric on the undirected biconnected graph for the measurement of distances using cyclic subgraphs and to use higher connectivity among the vertices in the biconnected graph.

## Closed trail distance in a biconnected graph without loops

In this section, a metric between the vertices in a biconnected graph without loops via a closed trail (circuit) will be defined.

### Closed trail distance in a undirected graph

**Definition 1**. *A graph is a k-closed trail connected graph (k-CT) if every two vertices lie on the closed trail (circuit) with a length* ≤ *k. A k-CT component of the graph is a maximal k-CT subgraph*.

**Definition 2**. *A* ∞-*CT graph is a graph where every two vertices lie on a closed trail of any length*.

**Definition 3**. *Let G* = (*V*, *E*) *be a graph. Let* $d_{ct}:V\times V\to R_{0}^{+}$ *be defined by the equation*

$$\begin{array}{c} d_{ct}\left(u,v\right)=min_{CT(u,v)\subseteq G}\left|CT\left(u,v\right)\right|, \end{array}$$

*where CT*(*u*, *v*) *is a closed trail that contains the vertices u, v. Then the function d*_*ct*_ *is called the closed trail distance* (*CT*-distance).

**Theorem 1**. *The CT-distance is a metric on the set V*.

*Proof*. We verify the properties of the metrics.

From Definition 3 it follows that *d*_*ct*_(*v*, *v*) = *min*_*CT*(*v*,*v*)∈*G*_|*CT*(*v*, *v*)|. The length of the shortest closed trail that started in a vertex *v* and ended in the same vertex is equal to 0. Then ∀*v* ∈ *V d*_*ct*_(*v*, *v*) = 0. And from the definition it follows that ∀*u*, *v* ∈ *V d*_*ct*_(*u*, *v*) ≥ 0 because the distance between *u* and *v* is the number of edges in the closed trail containing *u* and *v*.

Symmetry of the distance is obvious: ∀*u*, *v* ∈ *V d*_*ct*_(*u*, *v*) = *min*_*CT*(*u*,*v*)∈*G*_|*CT*(*u*, *v*)| = *d*_*ct*_(*v*, *u*) because *G* = (*V*, *E*) is a undirected graph.

The triangle inequality: ∀*u*, *v*, *z* ∈ *V d*_*ct*_(*u*, *v*) + *d*_*ct*_(*v*, *z*) ≥ *d*_*ct*_(*u*, *z*) is proved in a constructive way. Let *CT*(*u*, *v*) and *CT*(*v*, *z*) exist and let |*CT*(*u*, *v*)| = *d*_*ct*_(*u*, *v*) and |*CT*(*v*, *z*)| = *d*_*ct*_(*v*, *z*). In the biconnected graph ∀*u*, *v* ∈ *V* a closed path (*CP*(*u*, *v*)) exists. Then we have two possibilities for the closed trail which satisfy |*CT*(*u*, *z*)| = *d*_*ct*_(*u*, *z*). First: *d*_*ct*_(*u*, *v*) + *d*_*ct*_(*v*, *z*) = |*CP*(*u*, *v*)| + |*CP*(*v*, *z*)| ≥ |*CP*(*u*, *z*)| = *d*_*ct*_(*u*, *z*) which does not violate the triangle inequality. Second: if |*CP*(*u*, *v*)| + |*CP*(*v*, *z*)| < |*CP*(*u*, *z*)| then we can join *CP*(*u*, *v*) and *CP*(*v*, *z*) in the vertex *v* to one a closed trail *CT*(*u*, *z*) and *d*_*ct*_(*u*, *v*) + *d*_*ct*_(*v*, *z*) = |*CP*(*u*, *v*)| + |*CP*(*v*, *z*)| = |*CT*(*u*, *z*)| ≥ *d*_*ct*_(*u*, *z*) which does not violate the triangle inequality either.

**Fig 2:**
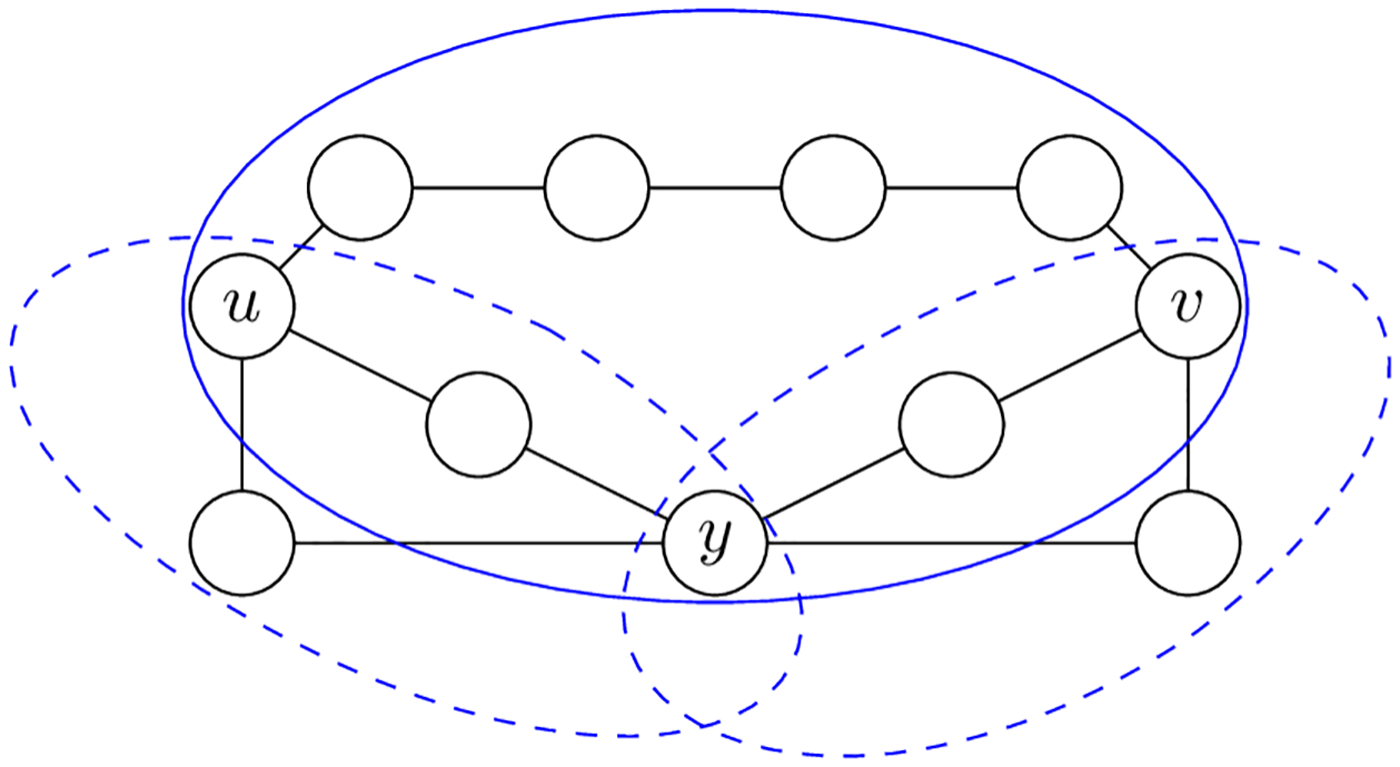
Example of biconnected graph where *d*_*sc*_(*u*, *y*) + *d*_*sc*_(*y*, *v*) < *d*_*sc*_(*u*, *v*).

**Example 1**. *Let graph G be defined as shown in* Fig 2.

*Let d*_*sc*_(*u*, *v*) *be the length of the shortest closed cycle in the biconnected graph that goes through the nodes u and v. The d*_*sc*_(*u*, *v*) *is not a metric, because it does not satisfy the triangle inequality*:

$$\begin{array}{c} d_{sc}\left(u,y\right)+d_{sc}\left(y,v\right)<d_{sc}\left(u,v\right). \end{array}$$

*For the graph in* Fig 2 *it is true that d*_*sc*_(*u*, *y*) = 4, *d*_*sc*_(*y*, *v*) = 4 *and d*_*sc*_(*u*, *v*) = 9.

*The violation of the triangle inequality by the cycles* (*d*_*sc*_) *may be solved using the closed trails* (*d*_*ct*_).

In a similar way to that in which we defined the *k*-CT component, a *k*-SC component may be defined using cycles of a length up to *k*. The graph is a *k*-shortest cycle connected graph (*k*-SC) if every two vertices lie on a cycle of the length ≤ *k*. The *k*-SC component of the graph is a maximal *k*-SC subgraph.

**Fig 3:**
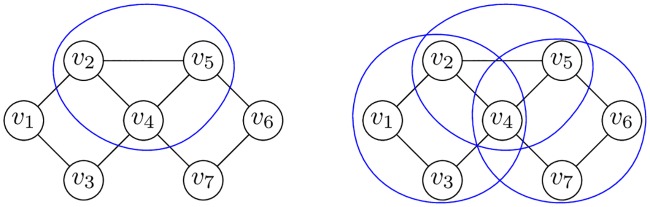
3-CT and 4-CT components of the graph.

**Example 2**. *Figs* 3 *and* 4 *demonstrate maximal and different k-CT components in the undirected biconnected graph without loops*.

**Fig 4:**
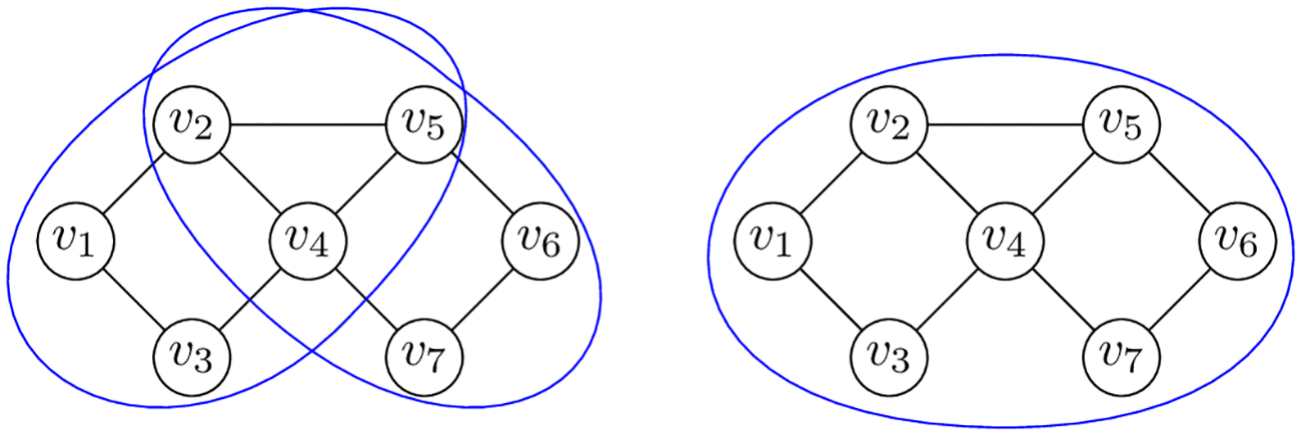
5-CT and 7-CT components of the graph.

**Lemma 1**. *Every 3-CT component is a clique*.

*Proof*. Let *Co*3 be a 3-CT component. According to Definition 3: ∀*u*, *v* ∈ *Co*3 |*CT*(*u*, *v*)| ≤ 3. It is obvious that this *CT*(*u*, *v*) contains only three vertices and three edges and there exists the edge (*u*, *v*). Therefore, all the vertices in *Co*3 are adjacent and create a clique.

**Fig 5:**
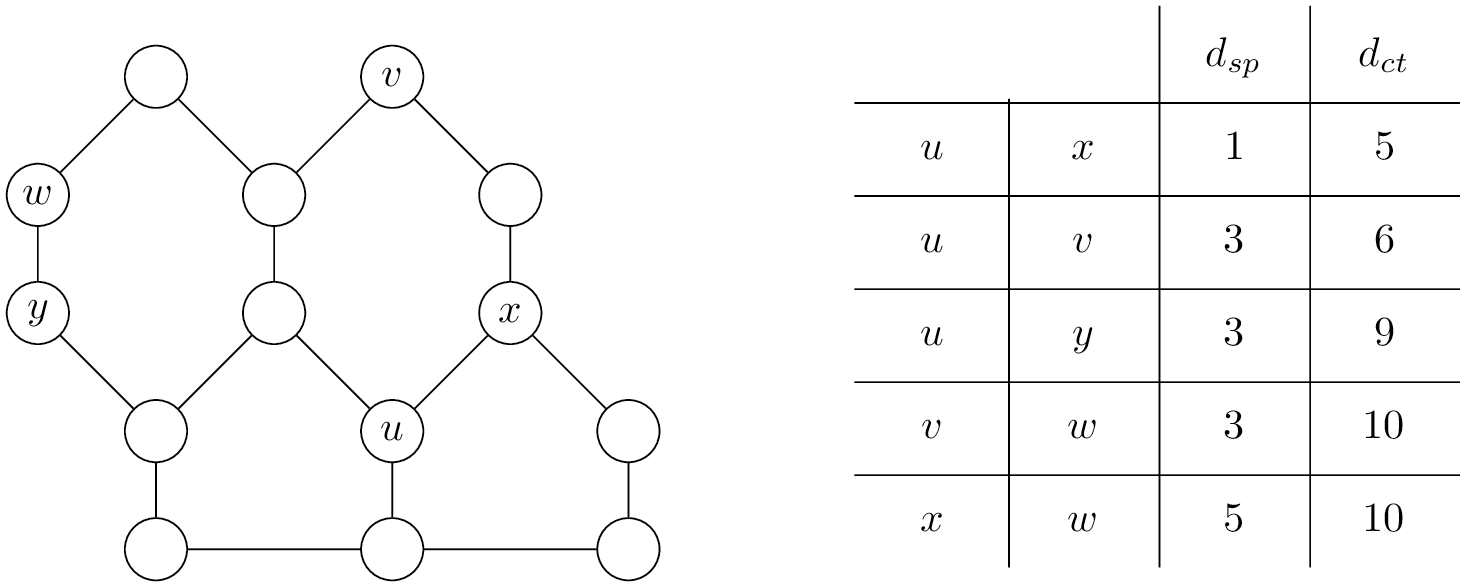
Selected vertices with the shortest path distance and the closed trail distance.

**Example 3**. Fig 5 *shows the differences between the closed trail distance and the shortest path distance. We chose a subgraph of the fullerene graph for the comparison between distances*.

**Lemma 2**. *Every closed trail with a length 4 or 5 is a cycle with the same length*.

**Fig 6:**
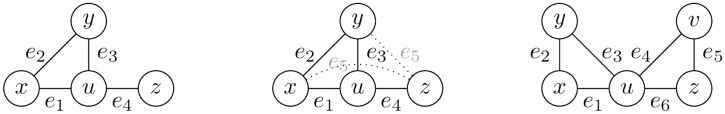
*u* is an initial and inner vertex in the trails T4 and T5. For the last closed trail CT6 is the vertex *u* initial, inner and final.

*Proof*. Proof by contradiction. Let there exist a closed trail with a length of 4 (*CT*4) or a closed trail with a length of 5 (*CT*5) which is not a cycle. Let *CP*3 be a cycle (closed path) with a length of 3. It is obvious that it is not possible to create *CT*4 (*CT*5) on the set with one, two or three vertices because *CT*4 (*CT*5) contains four (five) different edges. We have at least four vertices and *CT*4 (*CT*5) has to contain the vertex *u* which appears at least three times in the sequence of the trail (the initial, final and inner vertex of the closed trail). Vertex *u* is initial and inner vertex of the trail if *CT*4 (*CT*5) contain *CP*3. When we add one (two) edge(s) to the *CP*3 we have a trail with a length of 4 (5) but it not possible to create a closed trail because the edge between the last—but—one vertex and initial (final) vertex is already on the trail (Fig 6). The only closed trails with a length of 4 (5) are therefore cycles with a length of 4 (5).

The demonstration of the impossibility of creating a *CT*4 or *CT*5 which is not a cycle and the vertex *u* is the initial, inner but not final vertex of the closed trail with a length 4 (5):

$$\begin{array}{c} CT4=ue_{1}xe_{2}ye_{3}ue_{4}z,\;CT5=ue_{1}xe_{2}ye_{3}ue_{4}ze_{5}x\left(y\right). \end{array}$$

The first closed trail that is not a cycle is *CT*6 (Fig 6, third picture).

**Theorem 2**. *The 3,4,5-CT components are biconnected. They are the same as the 3,4,5-SC (shortest cycle) components*.

*Proof*. It is obvious for 3-CT components. The proof follows from Lemma 1. 3-CT components are cliques and therefore 3-CT components are biconnected.

**Fig 7:**
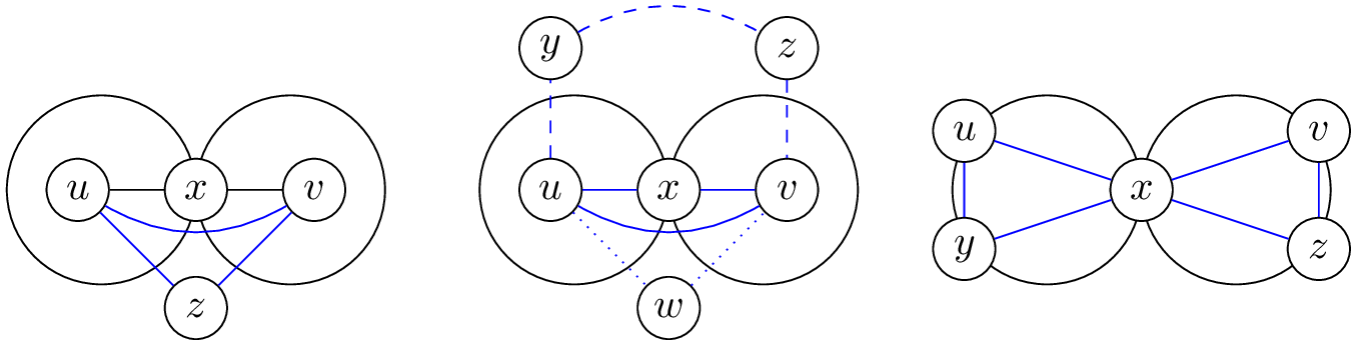
*k*-CT components. The first picture: *x* ∉ *CT*(*u*, *v*) and the component is biconnected. The second picture: *x* ∈ *CT*(*u*, *v*) where |*CT*(*u*, *v*)| = 3(4, 5) and the component is biconnected. The third picture: *x* ∈ *CT*(*u*, *v*) where this *CT* is the shortest closed trail which is not a cycle and the component is not biconnected.

We suppose that for 4(5)-CT components it is not true. There exists a connected 4(5)-CT component which is not biconnected. Then the component contains the articulation *x*—see Fig 7. Then there exist vertices *u*, *v*, where *d*_*ct*_(*u*, *v*) = 4(5). From Definition 3 it follows that there exist *CT*(*u*, *v*) and |*CT*(*u*, *v*)| = 4(5) (*CT*(*u*, *v*) is blue in Fig 7). From Lemma 2 it follows that all closed trails with a length of 4(5) are cycles and then the vertex *x* is not an articulation. This is a contradiction.

**Example 4**. *The k-CT component is not sometime a biconnected subgraph for k* ≥ 6. *The 6-CT component can be the smallest closed trail-connected component which is not biconnected* (Fig 6).

**Lemma 3**. *d*_*ct*_(*u*, *v*) *is a metric in any connected graph without bridges and defines the distances between two nodes u and v*.

**Fig 8:**
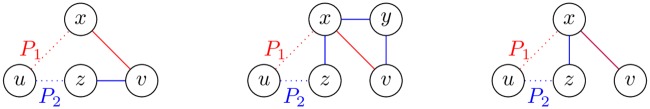
Path *P*_1_, *P*_2_ between vertices *u*, *v*. First graph is biconnected (*P*_1_ ∩ *P*_2_ = {*u*, *v*}), second graph is connected with articulation and without bridge (*P*_1_ ∩ *P*_2_ = {*u*, *v*, *x*}) and third graph is connected with bridge (*P*_1_ ∩ *P*_2_ = {*u*, *v*, *x*, (*x*, *v*)}).

*Proof*. The graph is connected without bridges. There is a path between any pair of nodes *u* and *v*. Let *P*_1_(*u*, *v*) = *ue*_(1)_ … *e*_(*i*)_ *xe*_(*i*+1)_ … *e*_(*k*)_*v* be a path between any pair of nodes *u* and *v*. Because the graph has no bridges (it is not 1-edge connected), a path *P*_2_(*u*, *v*) that has no common edge with *P*_1_(*u*, *v*) exists (see Fig 8). The joining of *P*_1_(*u*, *v*) and *P*_2_(*u*, *v*) creates a closed trail on which the nodes *u* and *v* lie (*CT*(*u*, *v*)). Therefore, a closed trail between any two nodes *u* and *v* with a length of *d*_*ct*_(*u*, *v*) may be created in any connected graph without bridges. The connected graph without bridges is a ∞ − *CT* component.

**Corollary 1**. *Graph and component hierarchy is defined as follows*:

$$\begin{array}{c} biconnected\;graph\subset connected\;graph\;without\;bridges=\infty -CT\;component. \end{array}$$

**Corollary 2**. *The ordering of type of components according to its cardinality is defined as follows*:

$$\begin{array}{c} 3-CT\subset 4-CT\subset 5-CT\subset \cdots \subset \infty -CT. \end{array}$$

**Fig 9:**
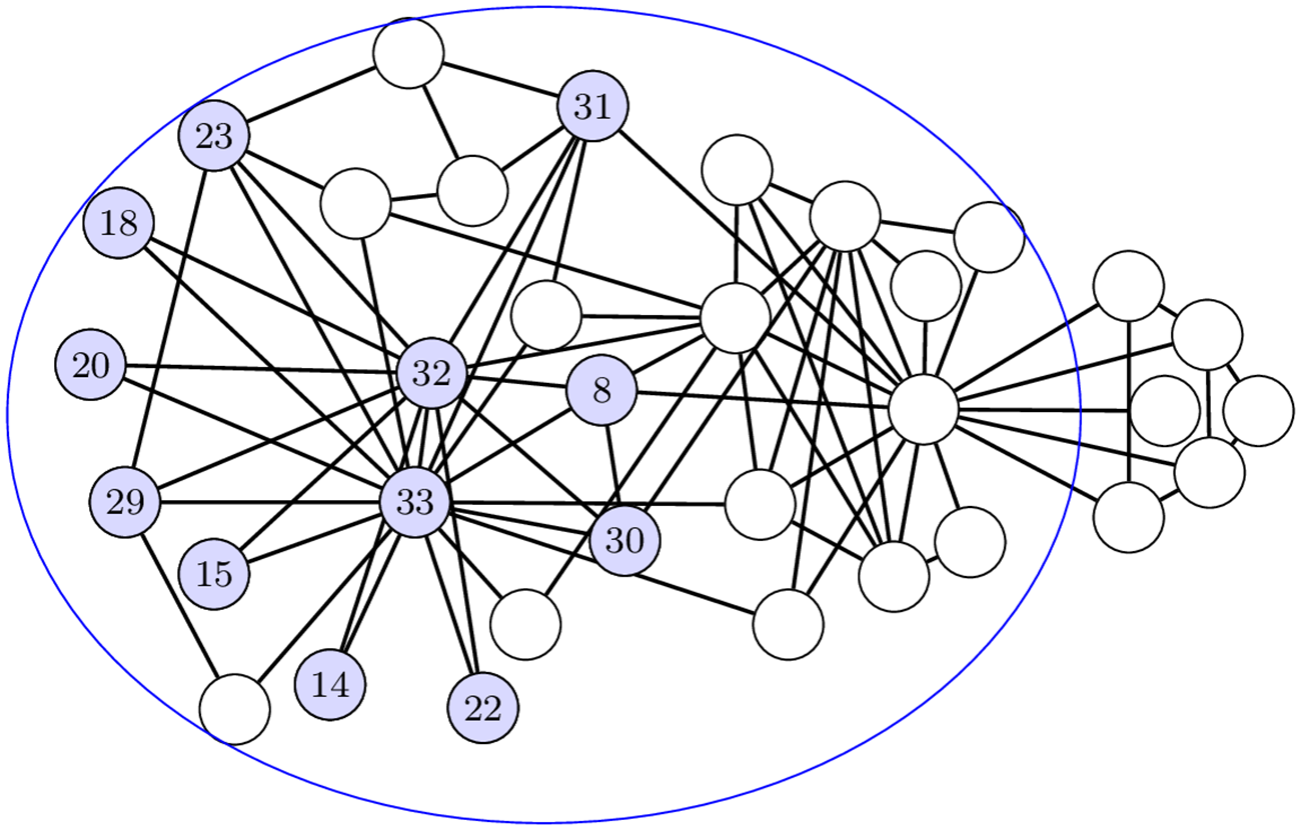
The largest 4-CT component (blue vertices) in the biconnected subgraph (in the blue ellipse) of Zachary’s karate club graph.

**Fig 10:**
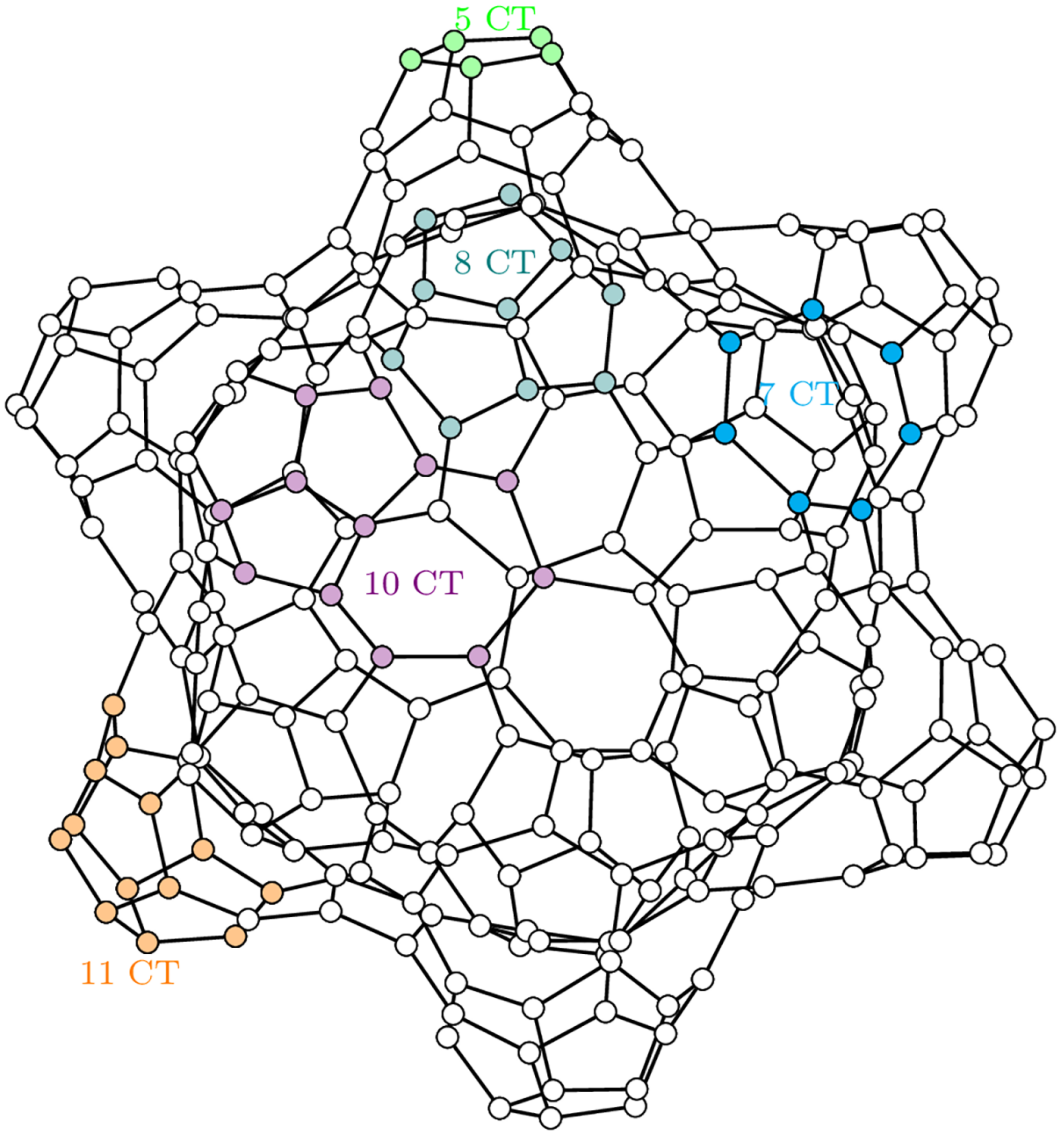
Graph of a fulleroid with a highlighted sample of the *k*-CT components.

**Example 5**. Fig 9 *shows the biggest 4-CT component (blue vertices) in the biconnected subgraph (in the blue ellipse) of the Zachary*’*s karate club graph* (Fig 9). *This 4-CT component is biconnected. The* Table 1 *contains numbers of k-CT components which are maximal for selected k. The* Fig 10 *shows the sample of k-CT components on the graph of fulleroid C*_260_ − *I*[5, 7].

**Table 1:** The number of different *k*-CT components in the largest biconnected subgraph of Zachary’s karate club graph.

	3-CT	4-CT	5-CT	6-CT	7-CT	8-CT	9-CT
no. of components	20	17	10	6	3	2	1

### Closed trail distance in a weighted undirected graph

The definition of the CT-distance may be extended for the weighted graph *G* = (*V*, *E*, *w*) where *w* is a mapping *w*: *E* → *R*^+^.

**Definition 4**. *Let G* = (*V*, *E*, *w*) *be a weighted graph and let the mapping* $d_{wct}:V\times V\to R_{0}^{+}$ *be defined by the equation*

$$\begin{array}{c} d_{wct}\left(u,v\right)=min_{CT(u,v)\subseteq G}(\sum_{\forall e\in CT(u,v)}\frac{1}{w(e)}). \end{array}$$

*Then the function d*_*wct*_ *is called the weighted closed trail distance (wCT-distance)*.

**Theorem 3**. *The wCT-distance is a metric on the set V*.

*Proof*. We verify the properties of the metrics.

From Definition 4 it follows that ∀*v* ∈ *V* $d_{wct}(v,v)=min_{CT(v,v)\in G}(\sum_{\forall e\in CT(v,v)}\frac{1}{w(e)})=0$ and ∀*u*, *v* ∈ *V* $d_{tw}(u,v)=min_{CT(u,v)\in G}(\sum_{\forall e\in CT(u,v)}\frac{1}{w(e)})\geq 0$.

Symmetry of the distance is obvious: $d_{wct}(u,v)=min_{\forall CT(u,v)\in G}(\sum_{\forall e\in CT(u,v)}\frac{1}{w(e)})=d_{wct}(v,u)$ because *G* = (*V*, *E*) is an undirected graph.

A triangle inequality ∀*u*, *v*, *z* ∈ *V* *d*_*wct*_(*u*, *v*) + *d*_*wct*_(*v*, *z*) ≥ *d*_*wct*_(*u*, *z*), is proved in a constructive way. Let *CT*(*u*, *v*) be a closed trail with the property that $\sum_{\forall e\in CT(u,v)}\frac{1}{w(e)}=d_{wct}(u,v)$ and let *CT*(*v*, *z*) be a closed trail with the property that $\sum_{\forall e\in CT(v,z)}\frac{1}{w(e)}=d_{wct}(v,z)$. Suppose that a closed trail *CT*(*u*, *v*, *z*) formed by the merged closed trail *CT*(*u*, *v*) and *CT*(*v*, *z*) exists. This closed trail has a weight of $w(CT(u,v,z))=\sum_{\forall e\in CT(u,v)}\frac{1}{w(e)}+\sum_{\forall e\in CT(v,z)}\frac{1}{w(e)}$. This equality implies that *d*_*wct*_(*u*, *z*) ≤ *d*_*wct*_(*u*, *v*)+*d*_*wct*_(*v*, *z*)

**Definition 5**. *The k-wCT component is a maximal subgraph H which satisfies* ∀*u*, *v* ∈ *V* *d*_*wct*_(*u*, *v*) ≤ *k*.

**Fig 11:**
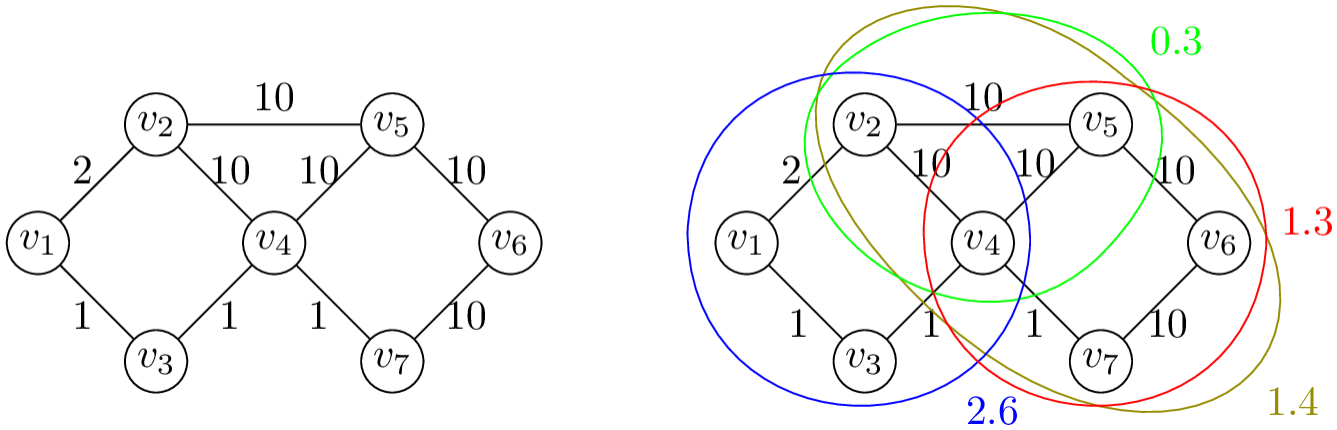
Weighted graph and selected wCT components of the graph.

**Example 6**. Fig 11 *demonstrate a different wCT components. A closed trail marked in blue CT* = *v*_1_ − (*v*_1_, *v*_2_) − *v*_2_ − (*v*_2_, *v*_4_) − *v*_4_ − (*v*_4_, *v*_3_) − *v*_3_ − (*v*_3_, *v*_1_) − *v*_1_ *has the weight* $\frac{1}{2}+\frac{1}{10}+\frac{1}{1}+\frac{1}{1}=\frac{13}{5}=2.6$. *It is the 4-CT component with the biggest weight. There exist 4-CT or 5-CT components with smaller weight* *(red*—{*v*_4_, *v*_5_, *v*_6_, *v*_7_} *and olive*—{*v*_4_, *v*_2_, *v*_5_, *v*_6_, *v*_7_}*)*. *The red marked closed trail contains edges with bigger weight than a blue marked closed trail. The value of the wCT component is useful for better scaling of components*.

## Conclusion

In this article, we defined a new metric for measuring the distance between two nodes of a biconnected graph. Moreover, the defined method holds the properties of a metric on the connected graphs without bridges. The same algorithm works on any connected graph, but it does not hold the metric properties. The metric reflects the cyclical interdependencies among the vertices of the graph. The metric derives components, called *k*-CT, the sets of vertices that have a distance between each pair of vertices less than or equal to *k*. The 3,4,5-CT components are biconnected because the closed trails with the length 3, 4, and 5 are cycles. The defined metric is applicable for both unweighted and weighted graphs.

The paper [34] contains a method for community detection based on network decomposition. Another approach to detection of overlapping communities is used by Palla et al. [35]. Both approaches use a clique percolation method [36] for community detection, more precisely, they use cliques for specifying part of a community. A clique is a 3-CT component according to our approach. Our proposed measure and a component definition decompose the graph into overlapping components using different *k*-CT components.
